# A Dynamic IIoT Framework Based on the Publish–Subscribe Paradigm

**DOI:** 10.3390/s23249829

**Published:** 2023-12-14

**Authors:** Ioan Ungurean, Nicoleta Cristina Gaitan

**Affiliations:** Faculty of Electrical Engineering and Computer Science, Stefan cel Mare University of Suceava, 720229 Suceava, Romania

**Keywords:** Industrial Internet of Things, framework, things, virtual environment, fog computing

## Abstract

The use of the Internet of Things (IoT) technologies and principles in industrial environments is known as the Industrial Internet of Things (IIoT). The IIoT concept aims to integrate various industrial devices, sensors, and actuators for collection, storage, monitoring, and process automation. Due to the complexity of IIoT environments, there is no one-size-fits-all solution. The main challenges in developing an IIoT solution are represented by the diversity of sensors and devices, connectivity, edge/fog computing, and security. This paper proposes a distributed and customized IioT (Industrial Internet of Things) framework for the interaction of things from the industrial environment. This framework is distributed on the fog nodes of the IIoT architecture proposed, and it will have the possibility to interconnect local things (with low latency) or global things (with a latency generated by the Internet network). To demonstrate the functionality of the proposed framework, it is included in the fog nodes presented in other paper. These fog nodes allow the integration of CANOpen networks into an IioT architecture. The most important advantages of the proposed architecture are its customizability and the fact that it allows decision operations to be carried out at the edge of the network to eliminate latency due to the Internet.

## 1. Introduction

The concept of the Internet of Things (IoT) represents a new paradigm that changes the way individuals interact with physical objects in everyday life [1]. It enables the integration of ubiquitous objects with the Internet, facilitating the delivery of new innovative services that have the potential to streamline and economize time and expenses in people’s daily routines [2]. The main idea of the IoT concept is to bring things from the real world into the virtual environment. In this virtual environment, physical things can interact with each other or humans through the Internet.

A subset of the IoT is the Industrial Internet of Things (IIoT) [3], which focuses on IoT technologies and principles in industrial and manufacturing environments. IIoT includes specific technologies such as industrial and machine-to-machine (M2M) communication technologies and fieldbuses, focusing on increasing productivity and reducing operational costs that enable the new business models. In industrial automation and control systems, communication is carried out throughout the fieldbuses. These specific fieldbuses improve efficiency, reliability, and predictability. Several fieldbus protocols are used in industry to enable communication and control of devices and equipment. PROFIBUS (Process Field Bus), Modbus, DeviceNet, EtherNet/IP, CANOpen, EtherCAT, and PROFINET are among the most significant fieldbus protocols. Various equipment and components used in industrial environments are connected in a fieldbus network, such as sensors, actuators, HMI (human–machine Interfaces), and measuring and analysis instruments [4]. In the case of an IIoT architecture, all these specific technologies must be included. In addition to the technologies specific to the industrial environment, an IIoT architecture includes other technologies such as cyber–physical systems, blockchain technology, fog computing, cloud computing, edge computing, and big data analytics [5]. The IIoT presents additional challenges, including the need to address issues such as low response time due to latency constraints, restrictions in available network bandwidth, resource-constrained devices, and the requirement of continuous service even in the absence of an internet connection, and new security challenges [6].

If we analyze IIoT solutions from the literature (such as the surveys [7,8,9,10,11,12]), we can see that they are specific to the application for which they were developed, and are dedicated only to certain communication technologies (fieldbuses). In order to address this problem and to be able to design and develop an IIoT solution with multi-communication protocols and multi-applications, we propose a dynamic IIoT framework for the interaction of objects/things in the virtual environment based on the publish–subscribe paradigm. The proposed IIoT framework is distributed on the fog nodes. Objects corresponding to things from the local environment, things from other fog nodes, or virtual things can be instantiated within a fog node. In addition, specific objects can be designed and developed to connect to cloud IoT solutions that enable long-term storage and big data analytics. These objects can interact locally through a published subscribed environment based on polymorphism, and remotely with objects on other fog nodes through a middleware protocol based on the published subscribed paradigm. Thus, locally, low latency to external events can be achieved. Furthermore, the objects can remotely interact with each other where the latency is influenced by the Internet network, which is the best effort type.

The IIoT framework proposed in this paper represents a structured architecture designed to facilitate the implementation of customizable IIoT solutions. The proposed framework allows the integration of devices (sensors, actuators, PLCs) connected to different fieldbuses through fog nodes. Fog nodes enable interaction in a secure virtual environment for data collection, analysis, and operational automation of the IIoT system. This paper proposes and presents a customizable and dynamic IIoT framework that allows the interaction of objects/things in a virtual environment. The proposed solution has several advantages, such as cost-effectiveness, latency reduction by processing data at the edge of the network, and data security in the industrial environment. Also, the framework allows connection to other IoT frameworks/platforms, which enables the use of the facilities provided by these IoT platforms.

This paper is structured as follows: in Section 2, we present related works for the IIoT solution presented in the specialized literature; Section 3 presents the architecture of the proposed IIoT framework; possible use cases of the proposed IIoT framework are presented in Section 4. Discussions related to the solution proposed are presented in Section 5, and the conclusions are highlighted in Section 6.

## 2. Related Works

With the introduction of the concept of the IoT into industrial applications [13], the concept of IIoT was defined. IIoT is a subset of IoT, and it consists of fieldbuses, sensors, actuators, robots, machines, appliances, business processes, and personnel [14,15]. IIoT has significant market potential, companies studying the market estimate that it will reach a market value of USD 106.1 billion in 2026, with an annual average growth of 6.7% from USD 88.2 billion in 2023 [16]. IIoT includes all concepts and technologies used by IoT [15], including fog computing [17]. Cisco Systems proposed the fog computing term [18] to define a new paradigm that extends cloud computing and cloud services at the edge of the local network. The main idea is to bring the cloud services (storage, computational resources, data processing, and device management) closer to the place where data are generated [17]. For the development of IIoT solutions based on fog computing, in the literature, several reference architectures are proposed, such as the one proposed by the OpenFog Consortium [19] (standardized by IEEE as the “1934–2018 IEEE Standard for Adoption of OpenFog Reference Architecture for Fog Computing”), the one proposed in [20] and organized on five layers, or the proposal [21] as an extension of an SDN (software-defined network) or NFV (network function virtualization) [22]. Other reference architectures proposed in the literature are those proposed in [23,24,25,26].

In [27], the author summarized the version of existing solutions using fog computing for IIoT applications. They identified the following research challenges: power consumption/energy efficiency, throughput/rate/capacity, latency, cache-enabled edge devices. They showed that fog computing can be an enabler for IIoT applications. In [28], a fog computing framework based on SDN that uses a dynamic fog-to-fog offloading mechanism is proposed. The authors focus on obtaining real-time communication between fog nodes, considering the quality of connections and minimizing bandwidth consumption and latency. A fog computing platform (FCP) reference architecture for IIoT is proposed in [29]. These architectures have a high degree of abstraction without details on how the devices are integrated into the system. Furthermore, for the industrial field, we must consider the requirements that must be fulfilled [5] regarding real time, data security, data integrity, latency, and response time to external events.

The main solutions for fog computing from the specialized literature that are analyzed in different reviews or survey papers [13,17,18,30,31,32,33,34,35,36,37,38,39,40,41] present the high-level parts of the fog nodes, such as security, the task scheduling, or resource scheduling when resources are limited; these papers are not focused on how data are processed within fog computing.

There are several IoT Cloud platforms on the market that can be used for integration into different IoT solutions. They provide a wide range of services such as long-term data storage or data analytics. ThingsBoard [42] is an IoT Cloud platform that allows the retrieval of data from an IoT environment, before analyzing and displaying it graphically. Data are retrieved through a gateway or directly to IoT devices via MQTT (message queuing telemetry transport), HTTP, and CoAP application protocols (the platform also provides APIs for these protocols). The IoT Azure IoT Hub [43] is also an IoT framework that allows data analysis in the cloud. The data are brought into the platform directly from IoT devices or through a Gateway via HTTP(S) and MQTT protocols. The same is true of the following IoT cloud frameworks: Google IoT Core [44], IBM Watson IoT [45], AWS IoT Core [46], Alibaba IoT [47], Oracle IoT [48], and Siemens MindSphere [49], Bosh IoT Hub [50], Cisco Kinetic [51], and Eclipse Hono [52]. All these platforms can receive data through the MQTT [53] and HTTPS protocols directly or through a gateway. Other protocols are supported (not all platforms), such as CoAP [54] (constrained application protocol), AMQP [54] (advanced message queuing protocol), and XMPP [54] (extensible messaging and presence protocol), but no platform supports DDS (distributed data service).

The advantage of the IIoT framework proposed in this paper is that it is integrated into an IIoT architecture of a fog node and allows the processing of data closer to where they are generated (meaning we can achieve a low latency). Also, it is highly customizable and extensible, meaning the possibility of developing new objects (objects for CoAP, AMQP, and XMPP middleware can be developed and used in the IIoT framework to connect in IoT Cloud platforms). Another advantage is DDS support, which has been designed to be used in real-time environments [55,56] (its full name is data distribution service for real-time systems).

## 3. The Proposed Framework Architecture

**Figure 1:**
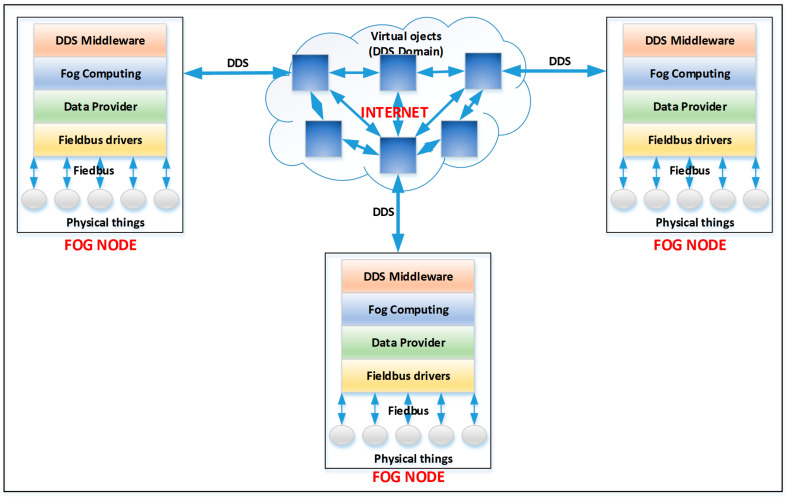
The general architecture of the proposed IIoT framework.

This paper proposes an IIoT framework that includes a virtual environment wherein objects/things can interact (exchanging data and updating their status, which can be reflected in the physical environment). The proposed framework is designed and developed as a collection of objects/things, each representing a specific physical thing (for example, temperature, pressure, etc.) or a virtual thing (for example, the average temperature over a certain amount of time). The general architecture of the proposed framework is presented in Figure 1. As can be seen, the architecture is geographically distributed (via the Internet), relying heavily on fog computing and edge computing concepts. Fog nodes are located at the edge of the network and connected to fieldbuses from the industrial environment (to which devices specific to the industrial environment are connected: actuators, transducers, PLCs, etc.).

The architecture of the IIoT framework is based on fog nodes that enable interaction in the virtual environment. Fog nodes perform data processing at the edge of the network, being able to react with low and predictable latency to events from the environment (which is very important for specific applications from the industrial environment). In addition, the nodes can interact with each other by exchanging data via the Internet. To apply this desideratum, fog nodes are developed around SoC (system-on-chip) systems that allow connection to fieldbuses by respecting real-time requirements and the line standard using specific peripherals (CAN, UART, etc.). The framework for object interaction is distributed within an IIoT network to fog nodes and other computing systems such as PCs, servers, etc. This functionality is achieved through the DDS protocol, creating a DDS domain wherein fog nodes can publish topics or subscribe to topics in the DDS domain.

**Figure 2:**
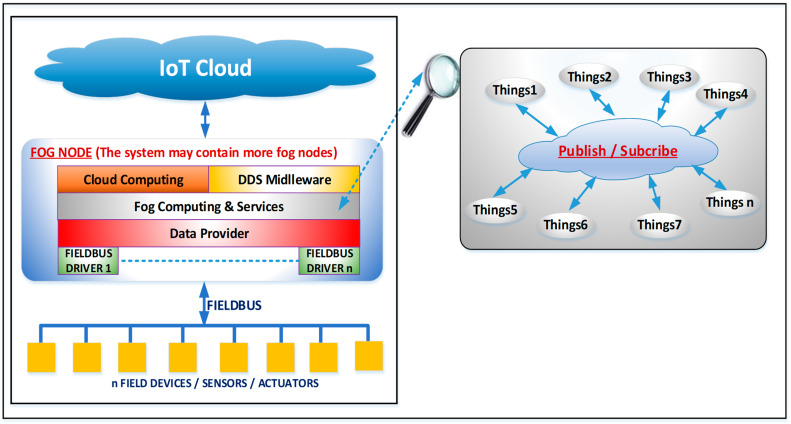
The virtual environment at the level of each fog node.

The software architecture of a fog node is shown in Figure 2. It can be seen that it includes a local virtual environment in which several objects can interact with each other. In this environment, a special object is instantiated for each fieldbus connected to the fog node. These special objects include all the data that can be acquired/set on the fieldbuses (specific to the industrial environment, such as the temperature acquired from a thermocouple, the pressure from a PLC, an analog output, etc.). The proposed solution is object-oriented. The objects are instantiated on fog nodes located at the edge of the local network. These objects can interact locally through a published subscribed environment based on polymorphism and remotely with objects on other fog nodes through a middleware protocol based on the published subscribed paradigm. Thus, we can achieve low latencies to local events on the fog node. Furthermore, objects can remotely interact with each other, but the latency is influenced by the Internet network, which is the best-effort type.

In this section, we present the IIoT framework that is included in fog nodes that implement the driver for the CANOpen fieldbus. The implementation of the driver for CANOpen fieldbus on a specialized SoC is described in detail in [57]. Each fog node will have its virtual environment in which objects from one or more fieldbuses (depending on the configuration) can be instantiated. In the local virtual environment, virtual objects can be created, which are published in the fog intra-node virtual environment through the DDS protocol. From Figure 2, it can be seen that there are two virtual environments: a local one, wherein the framework can respond to the events with low latency and perform edge/fog computing functions, and a global one, where the fog nodes exchange information between them (see Figure 1). This approach allows for better data protection because in the global virtual environment, only the data that the user has configured are published, avoiding security problems and possible flooding with unnecessary data of this environment.

**Figure 3:**
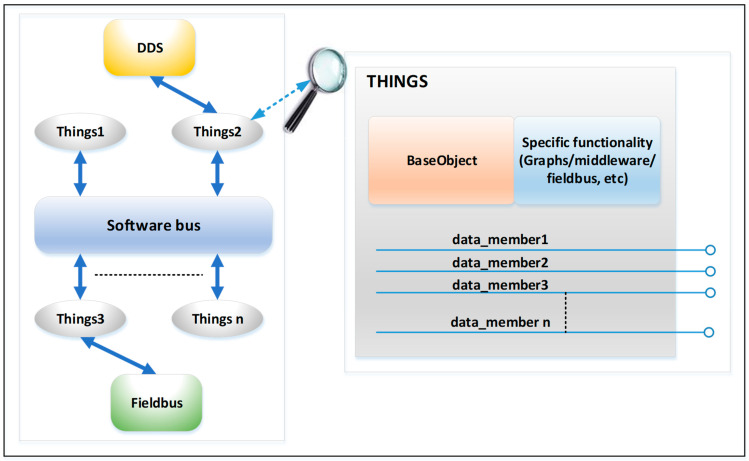
The general architecture virtual environment from each fog node.

Each object/thing has one or more data members/attributes (see Figure 3). Each attribute has as mandatory properties: data type (numeric, string, logical), access type (read-only—R, write only—W, or read–write R/W), a timestamp, and data quality (good, communication error, outdated, bad, etc.). In a publish/subscribe environment, objects/things will publish attributes that have the R or R/W access type. In this environment, objects/things can subscribe to one or more attributes and perform certain processing operations on the values (mathematical operations, mathematical functions, aggregation functions, etc.), and the result is associated with an attribute of the object/thinks (subscriber) that has the W or W/R access type. If this attribute has R/W as its access type, then the value is published in the virtual environment, and the other objects/things can subscribe to them (loop avoidance mechanisms will be included). Two types of things/objects can be created: physical things, which are associated with things defined at the data provider layer (things from the industrial environment), and virtual things, which can be defined at this level and represent the result of processing data from physical and/or virtual things. Virtual things may be graphical things for graphical data display; they may be things that can generate data or can bring data from other sources (via middleware systems, gateways, or other solutions). The data provider layer from the fog node (see Figure 2) will create an address space for industrial things/objects in the form of a collection of things/objects that the virtual environment uses. When a value is written to a property of a physical object/thing, this value is sent to the data provider layer and further to the corresponding fieldbuses (e.g., to a digital/analog output on the device associated with the object). A value read from the fieldbuses (e.g., a digital/analog input) is sent from the data provider layer to the corresponding object/thing in the virtual environment to be published and used by other objects/things.

There will be four types of virtual objects/things: expression, middleware, graphics, and others. Expression objects/things subscribe to one or more objects/things from the virtual environment, perform certain processing operations on the values (mathematical operations, mathematical functions, aggregation functions, etc.), and the result is published in the virtual environment.

The middleware objects/things allow the publication of data from the virtual environment through a middleware system (DDS—data distribution service [58,59], MQTT—message queuing telemetry transport [53], and the OPC UA server [60]) or the introduction of the data to a virtual environment through a middleware system (DDS, MQTT, and OPC UA client). The initial version will allow the instantiation of DDS objects (publishers and subscribers that can be used to interconnect fog nodes, including security specifications for DDS middleware protocol), MQTT objects/things (MQTT publishers for transferring data to other IoT platforms such as ThingsBoards [42]), or subscribing to other sources of data (there are many IoT things/devices/gateways that support MQTT middleware).

Graphic objects allow the graphical display of data from the virtual environment (forms, labels, graphic trends, bar graphs, images, etc.) or interaction with the user (buttons, input boxes, etc.). Through a configuration interface, we can instantiate objects in the virtual environment and configure forms (dashboards) and graphics elements displayed on forms (dashboards). These objects have as attributes their graphic properties (for example, color, background color, position on the dashboard, and visibility). These attributes can be connected in the virtual environment, and change their value dynamically (for example, the display color of a label may change if certain conditions are met).

Other objects are objects that do not fall into the above categories. Here, we have the historic object that can connect to a database to periodically save certain values from the virtual environment. This object allows the analysis of histories over a certain period of time. Another object is the alarm object that performs certain actions if certain alarm conditions are met.

Given the functionalities presented so far, the virtual environment will be in the form of software modules that run on fog nodes under Linux, and the access to the configuration and graphical display is performed remotely through a web server (from the browser, you can access the configuration part and the dashboards defined in the configuration step). Access to the node’s resources through the web server will be secured through a prior login to an interface that will acknowledge the user’s rights (there will be several levels of rights for users: admin, user, and guest).

Access to the virtual environment is implemented through a web server at the level of each fog node. The server can be accessed from a PC/smartphone/tablet in the same subnet as the fog node (it is behind a NAT (network address translation) for security reasons). Objects can be instantiated from this web server, connections between objects can be configured, and dashboards can be created to display graphic objects (for example, a digital display, a bar graph, etc.). Furthermore, through the web server, the DDS objects can be configured to publish data in the DDS domain or subscribe to data from the DDS domain, allowing interconnection with the other fog nodes.

## 4. Case Studies Description

To better understand the usefulness of the framework for interaction between objects and things and its potential value, we will present a simple example of usage. We consider that we have a CANOpen fieldbus to which two devices are connected. One device has eight digital outputs, and the other device has eight analog inputs (for example, a 4–20 mA current input to which we can connect pressure transducers). The fog node presented in [57] integrates the framework that was proposed within this paper.

**Figure 4:**
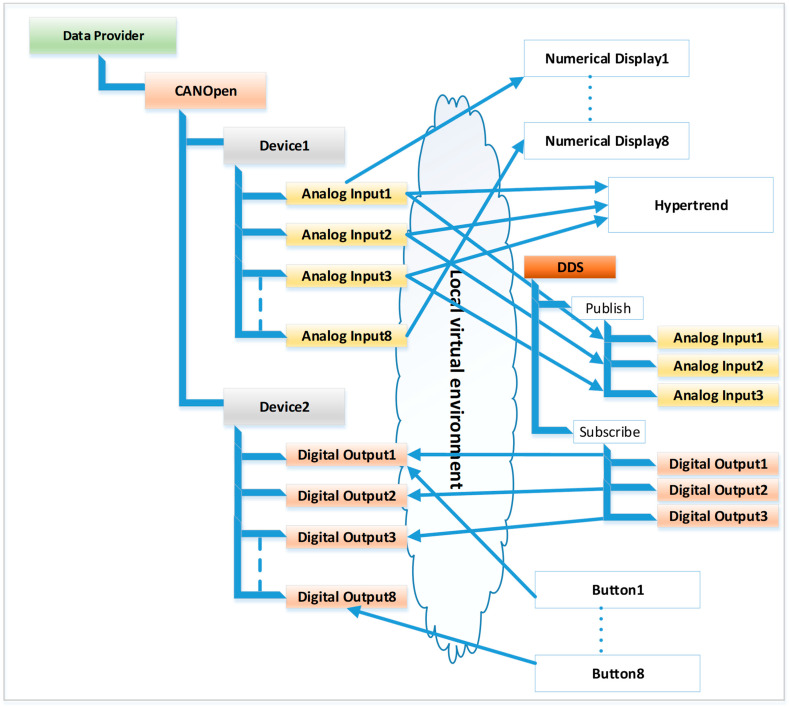
Local connection for the example of two devices connected to a CANOpen fieldbus.

**Figure 5:**
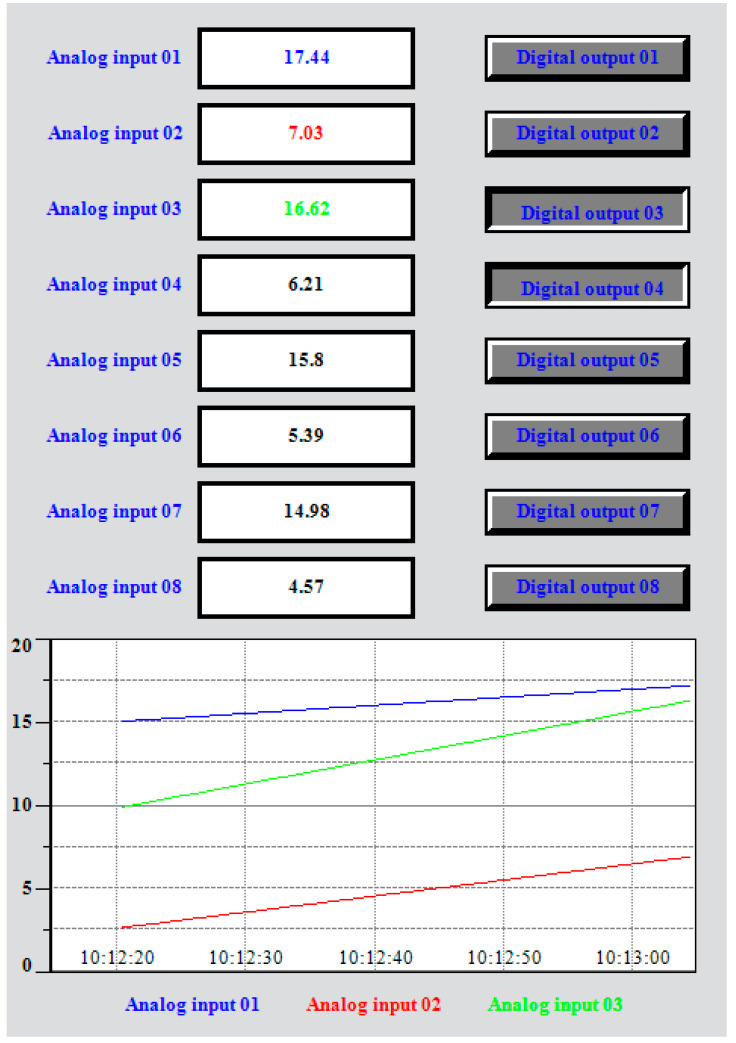
The dashboard for the example of two devices connected to a CANOpen fieldbus.

In the virtual environment, eight “number display”-type graphic objects are instantiated, and they are connected to the eight analog inputs provided by the data provider layer for the first device. Also, eight “button”-type objects are created, and they are connected to the eight digital outputs. In addition to these graphic objects, a HyperTrend-type object is created that connects to three analog inputs from the first device. Also, a DDS-type object is created that connects to three analog inputs (the data are published in the DDS domain) and to three digital outputs (subscribers to the DDS domain). Through this DDS object, local data are connected to the DDS domain, where they can be consumed or set by another fog node. The connections in this application are shown in Figure 4. All these graphic objects are displayed on a dashboard, an example being the one shown in Figure 5 (for each object, different graphic characteristics such as color, background, font, border, etc. can be set).

If a 4–20 mA current generator is connected to the analog input (for example, an Omega PCL1200 device) and the current at the device input is changed, the corresponding new value is displayed on the numerical display, and on the corresponding graph from the HyperTrend object. From the dashboard, if the button is pressed, then the respective action will be reflected in the digital output (which can command a multitude of actions in the industrial environment).

Another example of utilization can be the control and monitoring of a smart home. If the Modbus drivers are developed, then the IIoT framework can be used to monitor and view the parameters from the house (such as temperature and humidity), and can be configured to make certain decisions depending on these parameters (e.g., starting the heating system). These operations are performed on fog nodes, and depending on the configuration of the Internet connection or via a VPN, these data can be viewed remotely via a browser.

## 5. Discussions

The virtual environment consists of several software modules that run on fog nodes under Linux. Access to the configuration and graphical display is granted remotely through a web server (from the browser, you can access the configuration part and the dashboards defined in the configuration step). Access to the node’s resources through the web server is secured through a prior login interface that will acknowledge the user’s rights (there are several levels of rights for users: admin, user, guest).

The proposed IIoT framework is customizable and dynamic in the sense that it allows the instantiation of objects and their dynamic interaction in the virtual environment. Thus, specific applications can be created only by instantiating objects and configuring the interconnection of objects in the virtual environment without modifying software modules. In addition, the IIoT framework is extensible, because we can develop new types of objects. For example, we can develop an MQTT object to connect and transmit data to an IoT cloud.

Using DDS middleware to interconnect fog nodes allows real-time communication provided by DDS [58]. In addition, data security is provided by the DDS middleware, which includes authentication, encryption, access control, and data integrity mechanisms. Also, data security is achieved because not all data are published through DDS, allowing the user to select only the data required for this purpose.

If the data processing is carried out locally, then a low latency is achieved, meaning certain decisions can be made with low latency (and implicitly, a low response time) to external events taken from the industrial environment.

Starting from the use case from the previous section, for the case wherein a digital output is commanded (by pressing the “Digital Output 01” button), from the occurrence of the event to the sending the command on the CANOpen fieldbus, a maximum time of 321 µs was measured (with a jitter of 55 µs). The time was measured by using the clock_gettime() function when the two operations were handled in the software that implemented the IIoT framework. If the command is sent remotely from another fog node, we must add the time for transmitting the command on the Internet, where a good time is of the order of ms [61,62] (the Internet is a best-effort network). For this reason, operations that require a low latency must be performed locally, and the data can be sent to an IoT cloud platform (such as ThingsBoard [42]) for more advanced processing, such as big data analytics.

The potential of this framework for object interaction is very high, because it can be continuously developed and will snowball. In this paper, the functionality of this framework is validated and tested with a basic set of virtual objects, but it can be continuously developed by adding new graphic or middleware objects. As an example, if an object OPC UA client is developed, then the framework can operate as an OPC UA client for the graphical display of the data provided by OPC UA servers.

In addition, the local objects can be connected to an MQTT object to publish data to another IoT platform (such as ThingsBoard [42]) or to a historical object to record the evolution of these values over time. The ability to instantiate objects and the multitude of object interconnection options make this framework usable in a large number of scenarios. In addition, in the data provider layer, other drivers can be developed for other fieldbuses or other communication technologies such as LoRa, Sigfox, 5G, etc.

The solutions for fog computing from the specialized literature that are analyzed in different review or survey papers [13,17,18,30,31,32,33,34,35,36,37,38,39,40,41] specify that either they are presented at an abstract level or they are solutions for specific applications. An essential advantage of the proposed solution, which distinguishes it from the solutions presented in the specialized literature, is customizability, because it allows the instantiation of objects and their interconnection to implement specific solutions. Another advantage is extensibility, because they can develop new types of objects that implement new functionalities. In terms of disadvantages, we can acknowledge that it is linked to the Linux platform, and that drivers must be developed on SoC systems for each fieldbus integrated into the IIoT framework.

## 6. Conclusions

This paper proposes a distributed Industrial Internet of Things framework for the interaction of things from the industrial environment. This framework is distributed on the fog nodes of the IIoT architecture proposed, and has the potential to interconnect local things (with a low latency) or global things (with a latency generated by the Internet network).

The IIoT framework proposed in this paper proves that the Internet of Things can be used with field buses from industrial environments with the preservation of specific characteristics such as real time, robustness, predictability, integrity, safety, and security. One impact may be the possibility of treating unitary fieldbuses by using description methods that can be used to integrate fieldbuses in different systems, such as the case of a solution to the IIoT paradigm. Furthermore, the framework is customizable, and new objects can be added as needed. The solution proposed in this paper is suitable for automation and remote monitoring (the size of the monitored and controlled processes will be determined after the performance tests).

## Data Availability

No new data were created or analyzed in this study. Data sharing is not applicable to this article.
